# Author Correction: The immune response to RNA suppresses nucleic acid synthesis by limiting ribose 5-phosphate

**DOI:** 10.1038/s44318-024-00152-y

**Published:** 2024-06-21

**Authors:** Pushpak Bhattacharjee, Die Wang, Dovile Anderson, Joshua N Buckler, Eveline de Geus, Feng Yan, Galina Polekhina, Ralf Schittenhelm, Darren J Creek, Lawrence D Harris, Anthony J Sadler

**Affiliations:** 1 Centre for Innate Immunity and Infectious Diseases, Hudson Institute of Medical Research and Department of Molecular and Translational Sciences, Monash University, Clayton, VIC 3168 Australia; 2 Drug Delivery, Disposition and Dynamics, Monash Institute of Pharmaceutical Sciences, Monash University, Parkville, VIC 3052 Australia; 3 Ferrier Research Institute, Victoria University of Wellington, Lower Hutt, 5010 New Zealand; 4 Australian Centre for Blood Diseases, Department of Clinical Hematology, Monash University, Clayton, VIC 3004 Australia; 5 Department of Epidemiology & Preventive Medicine, Monash University, Melbourne, VIC 3004 Australia; 6 Monash Proteomics & Metabolomics Facility, Department of Biochemistry and Molecular Biology, Biomedicine Discovery Institute, Monash University, Clayton, VIC 3800 Australia

## Abstract

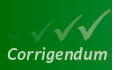

**Correction to:** *The EMBO Journal*. 10.1038/s44318-024-00100-w | Published online 22 May 2024

In this article, the author’s name ‘Feng Yan’ was incorrectly written as ‘Feng Alex Yan’. The original article has been corrected.

