# Supplementary material for: The immune response to RNA suppresses nucleic acid synthesis by limiting ribose 5-phosphate
Source: EMBO J. 2024 May 22;43(13):2636–60. doi: 10.1038/s44318-024-00100-w (PMC11217295; doi:10.1038/s44318-024-00100-w)
Supplement: Supplementary file 1 — Appendix [file 44318_2024_100_MOESM1_ESM.pdf]

SUPPLEMENTARY MATERIALS

The immune response to RNA suppresses nucleic acid synthesis by limiting ribose 5-phosphate.

Pushpak Bhattacharjee, Die Wang, Dovile Anderson, Joshua Buckler, Eveline de Geus, Feng Alex Yan, Galina Polekhina, Ralf Schittenhelm, Darren Creek, Lawrence Harris, Anthony Sadler.

Corresponding author: anthonysadler630@gmail.com

The file includes:

Appendix Supplementary Methods (P1 to 5)

Appendix Figures S1 to 11 (P1 to 17)

Appendix Table S1 (P18 to 19)

Appendix supplementary methods of the chemical synthesis

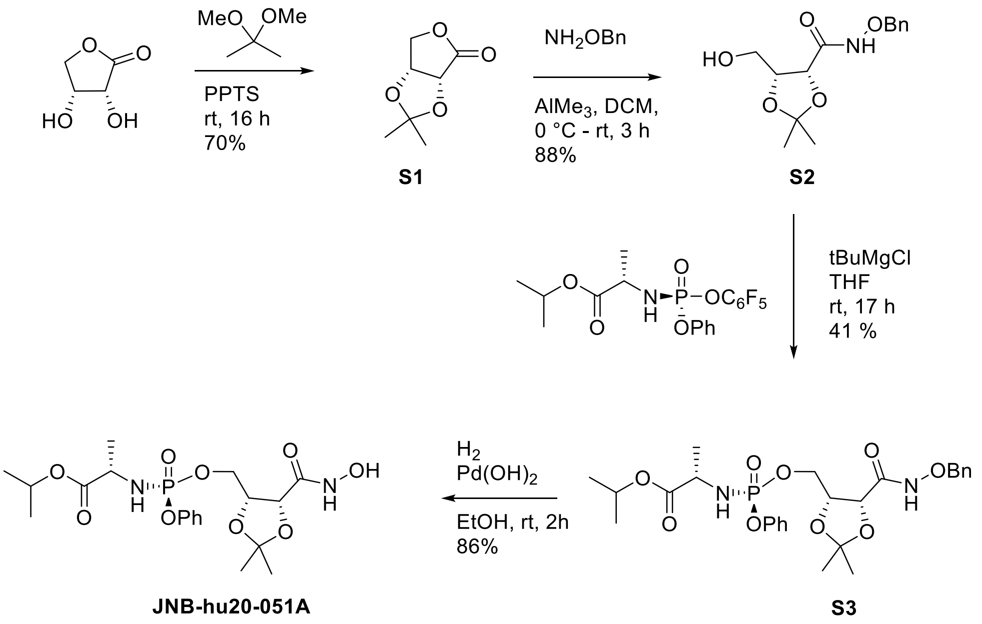

Appendix Figure S1

Synthesis of JNB-hu20-051A

*(3*aR,6aR*)-2,2-dimethyldihydrofuro[3,4-d] [1,3] dioxol-4(3aH)-one* S1 (2.80 g, 70 %) was prepared from D-erythronate-γ-lactone (3.00 g, 25.4 mmol) using established procedures (*97*);

*(4*R*,5*R*)-N-(benzyloxy)-5-(hydroxymethyl)-2,2-dimethyl-1,3-dioxolane-4-carboxamide* **S2**. Was prepared following procedures (*98*).

Preparation of NH_2OBn free-base: A magnetically stirred solution of *O*-benzyl hydroxylamine hydrochloride (3.26 g, 20.2 mmol) in dry methanol (30 mL), maintained at 0 °C under an atmosphere of argon, was treated with sodium methoxide (4.4 mL of a 25% w/v solution in MeOH, 19.6 mmol) and the solution was stirred for 1 hour at 0 °C. The mixture was then diluted with DCM (100 mL), filtered and concentrated under reduced pressure. The resulting residue was dissolved in hot ethyl acetate (20 mL), immediately filtered, and then concentrated under reduced pressure to afford *O*-benzyl hydroxylamine (2.41 g, 94) as a clear, colourless oil that was used immediately in the next step.

Lactone S1 (2.00 g, 12.6 mM) and *O*-benzyl hydroxylamine (2.41 g, 19.0 mM) were dissolved in dry DCM (135 mL) and stirred under an atmosphere of argon at room temperature for 0.5 h. The mixture was then cooled to 0 °C and trimethylaluminum (7 mL of a 2 M solution in toluene, 14 mM) was added dropwise over 5 minutes in a cooling bath, then removed and the mixture was stirred at room temperature for 3 h, after which time LCMS analysis indicated complete consumption of starting material. The mixture was poured into a beaker of ice-cold $NaHCO_3$ (200 mL of a saturated solution), and the biphasic mixture was stirred for 15 minutes before potassium sodium tartrate (200 mL of a saturated solution) was added and the mixture was stirred vigorously for 16 h. The biphasic mixture was then transferred to a separatory funnel, the organic layer separated, and the aqueous layer extracted with DCM (2 x 100 mL). The combined organic layers were washed with brine (100 mL), dried ($MgSO_4$), filtered and concentrated under reduced pressure to afford crude **3** as a viscous, colourless oil that solidified upon standing. This was subjected to chromatography on a Büchi Pure chromatography apparatus (silica, 1:9 v/v ethyl acetate/petroleum ether → ethyl acetate gradient elution). The concentration of the relevant fractions (R_f=0.3 in 1:3 v/v petroleum ether/ethyl acetate) afforded alcohol S2 as a white solid, 3.13 g (88%) yield. ^{1}H NMR (500 MHz, $CDCl_3$) δ 8.86 (s, 1H), 7.45–7.32 (m, 5H), 4.97 (d, *J*=11.4 Hz, 1H), 4.92 (d, *J*=11.3 Hz, 1H), 4.65 (d, *J*=7.6 Hz, 1H), 4.52 (ddd, *J*=7.6, 7.3, 4.6 Hz, 1H), 3.85–3.77 (br m, 1H), 3.66 (dd, *J*=12.1, 7.3 Hz, 1H), 3.12 (br s, 1H), 1.34 (s, 3H), 1.32 (s, 3H); MS (ESI, +ve) *m/z* 304.1 [$(M+Na)^+$, 30%], 483.2 (40), 224.1 (100); HRMS (ESI, +ve) *m/z* $(M+Na)^+$ calculated for $C_{14}H_{19}NO_5Na$ 304.1164, found 304.1161. These data match those reported in the literature (*98*).

*Isopropyl ((*S*)-(((4*R*,5*R*)-5-((benzyloxy)carbamoyl)-2,2-dimethyl-1,3-dioxolan-4-yl) methoxy) (phenoxy)phosphoryl)-*L*-alaninate* S3. A magnetically stirred solution of alcohol S2 (250 mg, 0.889 mmol) in dry THF (5 mL), maintained under an atmosphere of argon, was treated with a solution of *tert*-butyl magnesium chloride (2.5 mL of a 0.7 M solution in THF, 1.8 mmol) following established procedures (*97*). The clear solution was stirred at room temperature for 0.5 h, then isopropyl ((*S*)-(perfluoro phenoxy) (phenoxy)phosphoryl)-*L*-alaninate (600 mg, 1.32 mmol) was added in portions over 5 minutes. The resulting solution was stirred for 16 h before being quenched with isopropanol (2 mL) and concentrated under reduced pressure. This was then subjected to chromatography on a BUCHI Pure chromatography apparatus (silica, DCM → 3:7 v/v acetone/DCM gradient elution). The concentration of the relevant fractions (R_f=0.3 in 1:9 v/v acetone/DCM) afforded **S3** (200 mg, 41%) as a clear, colourless oil. $[a]_D^{25}$=+22.3 ($CHCl_3$, *c* 1.1); ^{1}H NMR (500 MHz, $CDCl_3$) δ 8.77 (s, 1H), 7.44–7.40 (m, 2H), 7.40–7.34 (m, 3H), 7.29–7.25 (m, 2H), 7.22–7.18 (m, 2H), 7.14–7.08 (m, 1H), 5.01 (hept, *J*=6.3 Hz, 1H), 4.61 (d, *J*=7.8 Hz,

1H), 4.57 (ddt, *J*=7.8, 5.4, 2.3, 1.3 Hz, 1H), 4.42 (ddd, *J*=11.3, 6.2, 2.5 Hz, 1H), 4.14 (ddd, *J*=11.3, 7.2, 5.4 Hz, 1H), 4.03 (ddt, *J*=15.8, 8.8, 7.1 Hz, 1H), 3.77 (br app t, *J*=10.3 Hz, 1H), 1.37 (d, *J*=7.1 Hz, 3H, overlapping), 1.36 (s, 1H), 1.31 (s, 3H), 1.23 (d, *J*=6.3 Hz, 3H), 1.22(6) (d, *J*=6.3 Hz, 3H); ^{13}C NMR (125 MHz, $CDCl_3$) δ 173.0 (d, $^4J_{CP}$=8.1 Hz), 166.3, 150.8 (d, $^3J_{CP}$=6.9 Hz), 134.8, 129.5, 129.2, 128.8, 128.6, 124.7, 120.3 (d, $^4J_{CP}$=4.8 Hz), 110.5, 78.6, 76.1 (d, $^3J_{CP}$=8.0 Hz), 74.8, 69.0, 64.9 (d, $^2J_{CP}$=4.9 Hz), 50.2, 26.4, 24.4, 21.7, 21.6 (d, $^4J_{CP}$=6.8 Hz), 21.1, 21.0; ^{31}P NMR (202 MHz, $CDCl_3$) δ 3.3; MS (ESI, +ve) *m/z* 573.2 [$(M+Na)^+$, 100%], 551.2 [$(M+H)^+$, 50%], 264.1 (65); HRMS (ESI, +ve) *m/z* $(M+Na)^+$ calculated for $C_{26}H_{35}N_2O_9NaP$ 573.1978, found 573.1973.

*Isopropyl ((*S*)-(((4*R*,5*R*)-5-(hydroxycarbamoyl)-2,2-dimethyl-1,3-dioxolan-4-yl) methoxy) (phenoxy)phosphoryl)-*L*-alaninate* JNB-hu20-051A.

20% w/w Pd $(OH)_2$/C (5 mg, 10 weight %) was added in one portion to a magnetically stirred solution of benzyl ether S3 (50 mg, 0.078 mmol) in dry ethanol (1 mL). A balloon of hydrogen was attached, and the solution was degassed three times before being placed under an atmosphere of hydrogen and stirred for 3 h at room temperature. The hydrogen atmosphere was purged and replaced with argon, then the solution was filtered through celite (CAUTION: pyrophoric), washed with EtOH (10 mL) and concentrated under reduced pressure. The residue thus obtained was subjected to chromatography on a BUCHI Pure chromatography apparatus (silica, 3:7 v/v acetone/DCM gradient → 8:2 v/v acetone/DCM gradient elution). The concentration of the relevant fractions (R_f=0.3 in 1:1 v/v acetone/DCM) afforded 1 (36 mg, 86%) as a clear, colourless oil. $[a]_D^{25}$=+11 ($CHCl_3$, *c* 0.1); ^{1}H NMR (500 MHz, $CDCl_3$) δ 8.94 (s, 1H), 7.33–7.29 (m, 2H), 7.24–7.20 (m, 2H), 7.15 (app t, *J*=7.2 Hz, 1H), 5.03 (hept, *J*=6.3 Hz, 1H), 4.75 (d, *J*=7.8 Hz, 1H), 4.57 (dddd, *J*=8.1, 3.3, 3.2, 3.0 Hz, 1H), 4.32 (ddd, *J*=11.3, 6.4, 3.3 Hz, 1H), 4.28 (ddd, *J*=11.3, 4.5, 3.2 Hz, 1H), 4.01–3.87 (m, 2H), 1.50 (s, 3H), 1.37 (d, *J*=3.9 Hz, 3H, overlapping), 1.36 (s, 3H), 1.24 (d, *J*=6.3 Hz, 3H), 1.23 (d, *J*=6.3 Hz, 3H); ^{13}C NMR (125 MHz, $CDCl_3$) δ 173.3 (d, $^4J_{CP}$=6.7 Hz), 165.8, 150.6 (d, $^3J_{CP}$=6.7 Hz), 129.7, 125.0, 120.2 (d, $^4J_{CP}$=5.3 Hz), 110.6, 76.0 (d, $^3J_{CP}$=9.1 Hz), 74.8, 69.5, 65.3 (d, $^2J_{CP}$=5.8 Hz), 50.2, 26.4, 24.5, 21.7, 21.6, 21.0 (d, $^4J_{CP}$=5.5 Hz); ^{31}P NMR (202 MHz, $CDCl_3$) δ 2.5; MS (ESI, +ve) *m/z* 943.3 [$(2M+Na)^+$, 100%], 944.3 (40), 483.2 [$(M+Na)^+$, 60%]; HRMS (ESI, +ve) *m/z* $(M+Na)^+$ calculated for $C_{19}H_{29}N_2O_9NaP$ 483.1508, found 483.1509.

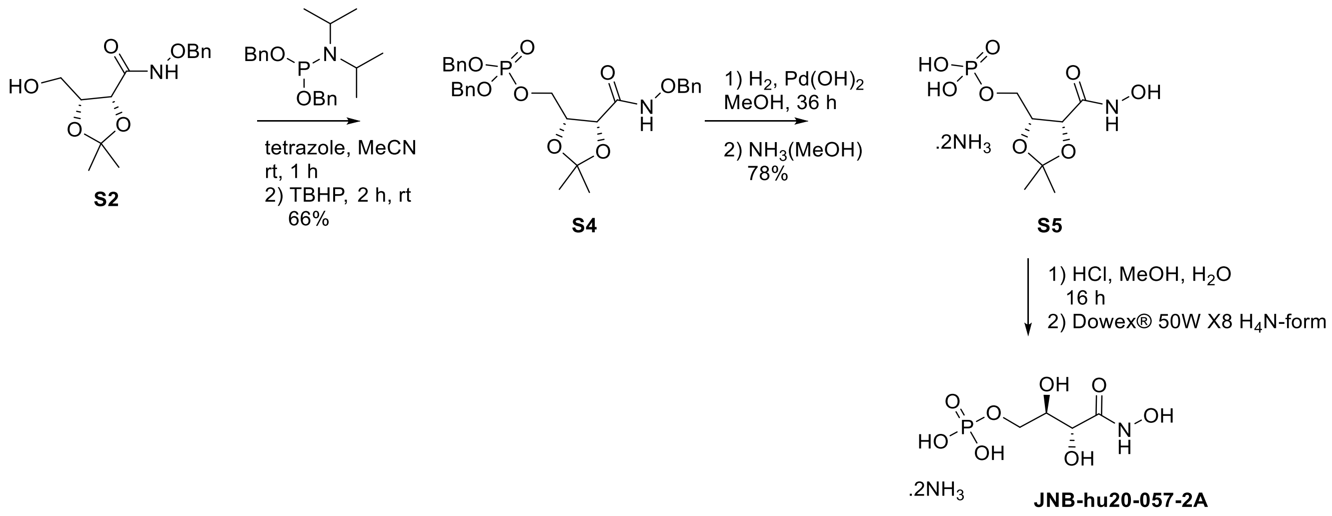

Appendix Figure S2

Synthesis of JNB-hu20-057-2A

*Dibenzyl (((4*R*,5*R*)-5-((benzyloxy)carbamoyl)-2,2-dimethyl-1,3-dioxolan-4-yl) methyl) phosphate* S4. Following established procedures (*99*), Dibenzyl diisopropyl phosphoramidite (1.0 mL, 2.8 mmol) was added dropwise over 15 minutes to a magnetically stirred solution of alcohol **3** (570 mg, 2.03 mmol) and 1H-tetrazole (10 mL of a 0.45 M solution in MeCN, 4.5 mmol) in dry acetonitrile (10 mL). The reaction was stirred for 1 h at room temperature, after which TLC indicated full conversion of the starting material to the intermediate phosphite. The solution was cooled to 0 °C and treated with *tert*-butyl hydroperoxide (1.7 mL of a 70% w/v solution in water, 12 mmol) then the cooling bath was removed, and the solution stirred at room temperature for 2 h. The mixture was diluted with deionized water (80 mL), extracted with DCM (3 x 100 mL) and the combined organic extracts were washed successively with $NaHCO_3$ (20 mL of a saturated solution) and brine (20 mL) before being dried ($MgSO_4$) and concentrated under reduced pressure. The residue thus obtained was subjected to chromatography on a BUCHI Pure chromatography apparatus (silica, petroleum ether → 7:3 v/v ethyl acetate/petroleum ether gradient elution). The concentration of the relevant fractions (R_f=0.2 in 1:1 v/v petroleum ether/ethyl acetate) afforded phosphate 5 (1.01 g, 66%) as a clear, colourless oil. ^{1}H NMR (500 MHz, $CDCl_3$) δ 8.78 (s, 1H), 7.41–7.29 (m, 15H), 5.08–5.04 (m, 4H), 4.92 (d, J=11.1 Hz, 1H), 4.87 (d, J=11.1 Hz, 1H), 4.58 (d, J=7.6 Hz, 1H), 4.52 (tdd, J=7.6, 2.7, 1.0 Hz, 1H), 4.38 (ddd, J=11.3, 6.7, 2.7 Hz, 1H), 4.05 (dt, J=11.3, 6.7 Hz, 1H), 1.39 (s, 3H), 1.30 (s, 3H). ^{31}P NMR (202 MHz, $CDCl_3$) δ -1.3, MS (ESI, +ve) m/z 542.2 [$(M+H)^+$, 100%], 264.1 (50), 543.2 (30). These data match those reported in the literature (*98*).

*((4*R*,5*R*)-5-(hydroxy carbamoyl)-2,2-dimethyl-1,3-dioxolan-4-yl) methyl dihydrogen phosphate diammonium salt* S5. 20% w/w Pd $(OH)_2$/C (84 mg, 42 weight %) was added in one portion to a magnetically stirred solution of benzyl ether S4 (200 mg, 0.369 mmol) in dry methanol (20 mL). A balloon of hydrogen was attached, and the solution was degassed before being placed under an atmosphere of hydrogen and stirred for 3 h at room temperature. The hydrogen atmosphere was replaced with argon, then the solution was filtered through celite, washed with methanol (20 mL), treated with methanolic ammonia (2 mL of a 1 M solution, 2 mmol), and then concentrated under reduced pressure to afford crude S5 (80 mg, 80%) as a light brown powder. Whilst the

bulk material was used directly in the next step without purification, a sub-sample (20 mg) was purified using a Sep-Pak C18 cartridge (eluting with deionized water) and the product-containing fractions were combined and lyophilized to afford pure **6** as a white powder (18 mg, 90% recovery). ^{1}H NMR (500 MHz, MeOD) δ 4.61–4.53 (m, 2H), 4.00 (dt, *J*=10.9, 5.4 Hz, 1H), 3.88 (dt, *J*=10.9, 5.6 Hz, 1H), 1.55 (s, 3H), 1.35 (s, 3H); ^{13}C NMR (125 MHz, MeOD) δ 168.3, 111.6, 78.3 (d, $^{2}J_{CP}$=9.7 Hz), 76.6, 64.72 (d, $^{3}J_{CP}$=4.3 Hz), 27.1, 25.2; ^{31}P NMR (202 MHz, MeOD) δ 1.5; MS (ESI, –ve) *m/z* 270.1 [(M–2NH$_3$–H)$^-$, 100%], 541.1 [(2(M–2NH$_3$)–H)$^-$, 50%], 113.1 (25). These data match those reported[1] in the literature.

*(2*R*,3*R*)-2,3-dihydroxy-4-(hydroxyamino)-4-oxobutyl dihydrogen phosphate diammonium salt* JNB-hu20-057-2A. A magnetically stirred solution of acetonide **S5** (60 mg, 0.20 mmol) in a mixture of MeOH and deionized water (1:1 v/v, 3 mL) was treated with one drop of concentrated HCl (*ca* 0.05 mL, 0.6 mmol). The mixture was stirred at room temperature for 16 h, after which time LCMS analysis showed the reaction to be complete. As the phosphoric acid proved to be unstable in our hands, it was converted to the diammonium salt: Triethylamine was then added until the pH was >7 (*ca.* 0.1 mL) and the mixture was concentrated under reduced pressure. Toluene was added (5 mL) and the mixture was concentrated again, before being dried at 0.1 mbar for 5 hours. The di-triethylamine salt intermediate was dissolved in deionized water (2 mL) and passed through an ion exchange column (1g Dowex® 50W X8 NH$_4$-form), eluting with deionized H_2O. The product-containing fractions were combined and lyophilized to afford the title compound (24 mg, 46%) as a white powder (contaminated with *ca* 5% compound **6** by ^{1}H NMR). ^{1}H NMR (500 MHz, D_2O) δ 4.29 (d, *J*=5.9 Hz, 1H), 4.09–3.97 (m, 3H); ^{13}C NMR (125 MHz, D_2O) δ 170.5, 71.5 (d, $^{2}J_{CP}$=6.7 Hz), 70.4, 64.4(d, $^{3}J_{CP}$=4.8 Hz); ^{31}P NMR (202 MHz, D_2O) δ 3.1; MS (ESI, –ve) *m/z* 461.0 [2(M–2NH$_3$)–H)$^-$, 100%], 230.1 [(M–2NH$_3$–H)$^-$, 95%], 113.1 (46). These data are consistent with those reported in the literature for the parent phosphoric acid (*98*).

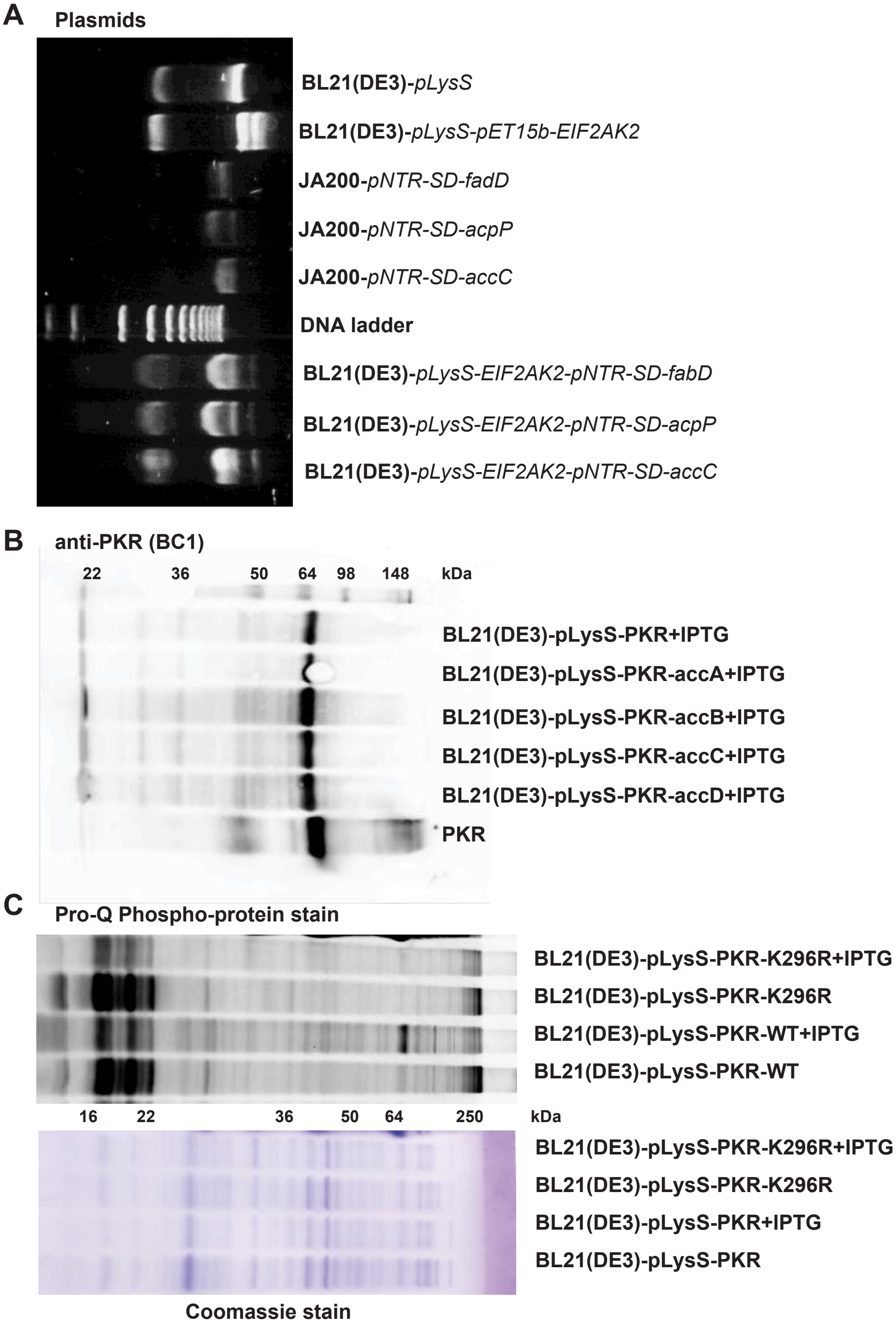

Appendix Figure S3

Supporting material for the *E. coli* complementation assay. (**A**) An agarose gel visualising plasmid DNA that was isolated from *E. coli*, then electrophoretic separated and stained with cyanine dye (SYBR Safe). The experiment shows isolation of plasmids for the T7 lysozyme

(*pLysS*, 4886bp), PKR (*pET15b-EIF2AK2*, 7364 bp) and three metabolic enzymes as mobile genetic elements (*pNTR-SD-FadD*, 9230 bp, -*AcpP*, 8537 bp and -*AccC*, 9650 bp) alone from the separate BL21(DE3) and JA200 strains and together in the recipient BL21(DE3) transformed with *pET15b-EIF2AK2* and mated with the F+ JA200 donors. (**B**) An immunoblot confirming the expression of human PKR in *E. coli* BL21(DE3)-pLysS transformed with *pET15b-EIF2AK2* and conjugated with F+ JA200 donor strains transformed with the indicated mobile genes and induced with IPTG by probing with a primary anti-PKR antibody (BC1) and secondary fluorescent anti-rabbit antibody. A purified preparation of recombinant PKR is run as a comparison (PKR). (**C**) Visualising PKR kinase activity in *E. coli* BL21(DE3)-pLysS-*pET15b-EIF2AK2* expressing the WT or kinase-dead (K296R) PKR, either untreated or induced with IPTG then lysed and separated by SDS-PAGE, before staining with dyes for phosphorylated (Pro-Q Diamond, at top) and total proteins (Coomassie, at bottom). Autophosphorylated PKR appears as a 68 kDa band in lysates from IPTG-induced bacteria expressing the WT kinase. Notably, proteins phosphorylated by bacterial kinases are also detected, including a 27 kDa product that is coincident with a phosphoprotein detected in **Fig. 5A**.

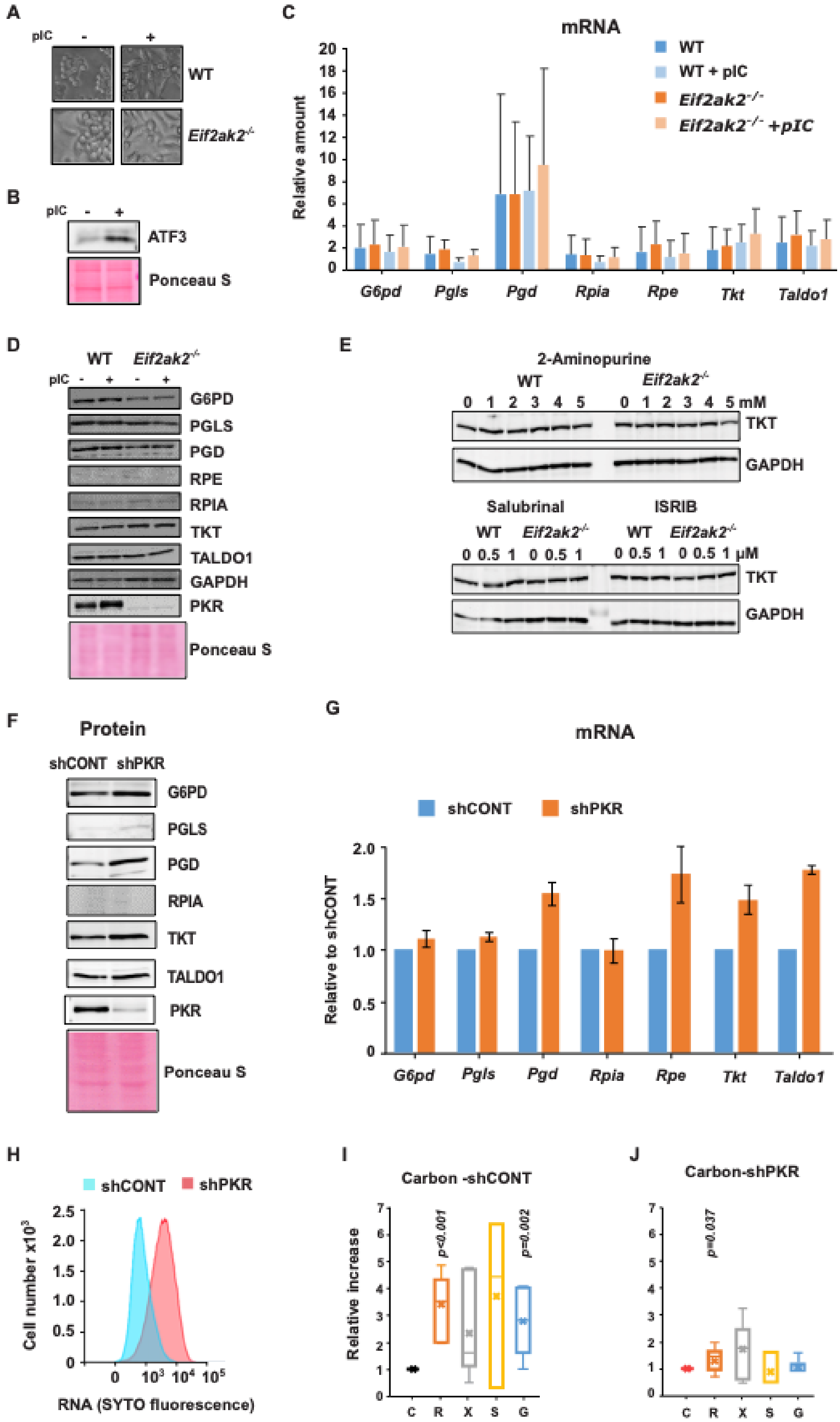

A
pIC - +
WT
Eif2ak2-/-
B
pIC - +
ATF3
Ponceau S
C
mRNA
WT
WT + pIC
Eif2ak2-/-
Eif2ak2-/- +pIC
Relative amount
G6pd Pgls Pgd Rpia Rpe Tkt Taldo1
D
WT Eif2ak2-/-
pIC - + - +
G6PD
PGLS
PGD
RPE
RPIA
TKT
TALDO1
GAPDH
PKR
Ponceau S
E
2-Aminopurine
WT Eif2ak2-/-
0 1 2 3 4 5 0 1 2 3 4 5 mM
TKT
GAPDH
Salubrinal ISRIB
WT Eif2ak2-/- WT Eif2ak2-/-
0 0.5 1 0 0.5 1 0 0.5 1 0 0.5 1 μM
TKT
GAPDH
F
Protein
shCONT shPKR
G6PD
PGLS
PGD
RPIA
TKT
TALDO1
PKR
Ponceau S
G
mRNA
shCONT shPKR
Relative to shCONT
G6pd Pgls Pgd Rpia Rpe Tkt Taldo1
H
shCONT shPKR
Cell number x10³
RNA (SYTO fluorescence)
I
Carbon -shCONT
Relative increase
p<0.001
p=0.002
C R X S G
J
Carbon-shPKR
p=0.037
C R X S G

Appendix Figure S4

Supporting material for assays in macrophages. (**A and B**) Measures of the response of the splenic WT and *Eif2ak2*$^{-/-}$ macrophages to polyinosinic-polycytidylic acid (pIC) by; (**A**) visualising the changed phenotype of the cells in culture and (**B**) detecting the induction of the Activating transcription factor 3 (ATF3) by immunoblot and Ponceau S stain. (**C**) Measures of the relative levels of PPP transcripts in WT and *Eif2ak2*$^{-/-}$ splenic macrophages by Q-PCR (n=3). The level of each transcript is shown normalized to that in the WT cells in **Fig. 3B**. (**D**) Representative measures of the PPP proteins in the WT and *Eif2ak2*$^{-/-}$ splenic macrophages by immunoblot with the indicated antibodies. A quantitation of protein levels from multiple immunoblots is shown in **Fig. 3C**. (**E**) Immunoblots of the levels of TKT in spleen-derived macrophages treated with polyinosinic-polycytidylic acid and the indicated modulators of the PKR response. The molecules work as follows; 2-aminopurine (2AP) inhibits PKR's kinase activity, ISRIB overrides the suppression of EIF2B by EIF2α phosphorylation and Salubrinal inhibits the EIF2α phosphatases. (**F and G**) The expression of PPP factors in the bone marrow-derived macrophages expressing either non-targeting (shCONT) or *Eif2ak2*$^{-/-}$targeting (shPKR) shRNAs by measures of; (**F**) proteins by immunoblot with the indicated antibodies and (**G**) transcripts by Q-PCR (n=3). (**H**) A representative FACS plot of SYTO RNASelect fluorescence in the shCONT and shPKR bone marrow-derived macrophages. (**I and J**) The relative cell counts of the (**I**) shCONT or (**J**) shPKR macrophages after culture without the addition of saccharides (C) compared to supplemented with; D-sedoheptulose (S) (n=3), D-ribose (R), D-xylose (X), L-arabinose (A) or glycerol (G) alone or combined (n=5). This data shows the effects of the different saccharides on each cell as an alternative to the presentation in **Fig. 2I and J**, which shows the effect of PKR on saccharide use. The data report the $\bar{x} \pm \sigma$. *P* values were calculated by the student's t-test of independent experiments.

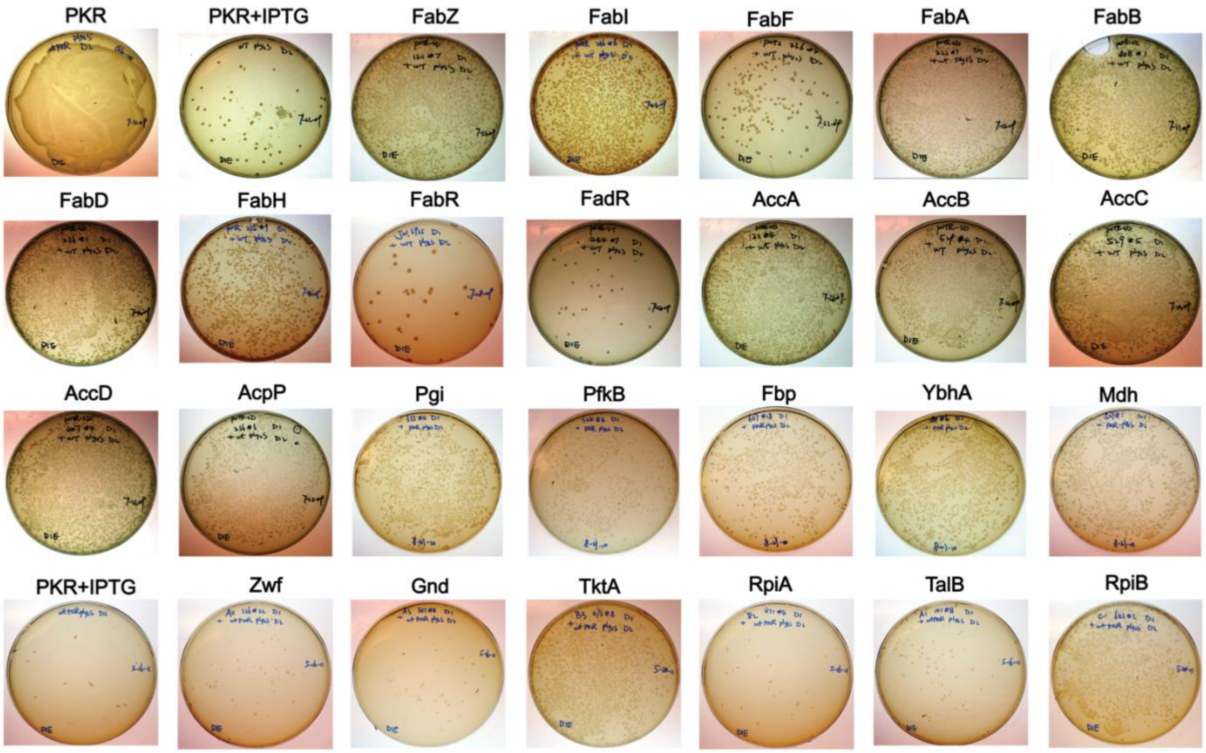

Appendix Figure S5

Supporting material for genetic complementation of transformed *E. coli*. Images of bacterial colonies on Luria-Bertani agar plates arising from mattings of the transformed *E. coli* strain BL21(DE3)-pLysS-*pET15b-EIF2AK2* with F+ JA200- *pNTR-SD* strains carrying the indicated *E. coli* metabolic factors as mobile genetic elements. All plates were treated with IPTG to induce PKR expression apart from the control (PKR, top left) and are supplemented with antibiotics to select each plasmid. Representative images of all mattings that rescued bacterial growth are shown along with some conjugations that didn't rescue as a comparison. All *E. coli* genes tested, with the full titles of the metabolic factors are listed in **Appendix Table S1**. Confirmation of the transfer of the mobile genetic elements and the induction of PKR expression and activity are shown in **Appendix Figure S3**. The panels showing the effect of the PPP enzymes Zwf, TktA and RpiA or the alternative isomerase RpiB on the PKR-induced auxotrophy are shown in **Fig. 3F**. This experiment is repeated with liquid cultures of *E. coli* expressing PKR with Zwf, Rpe, RpiA, TktA, TktB or RpiB in **Fig. 3G**. Notably, the TalB construct used in this experiment was mutated and so didn't capture enzyme activity but the activity of the human transaldolase is captured in **Fig. 4A and B**.

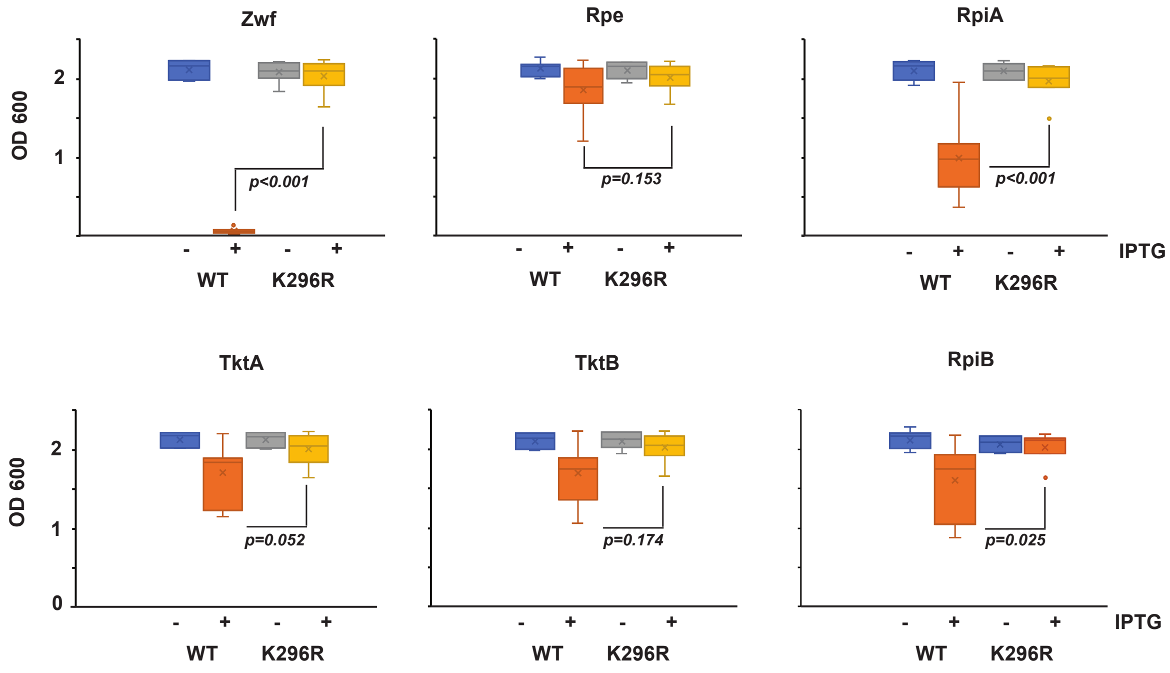

Appendix Figure S6

Supporting material for PKR's regulation of the *E. coli* PPP enzymes. Plots of the growth of *E. coli* BL21(DE3)-pLysS-*pET15b-EIF2AK2* expressing WT or kinase-dead (K296R) PKR alone or co-transfected with *pWKS130* expressing the indicated PPP genes from *E. coli* by measures of the optical density of the cultures (at 600 nm). The data shows the matched kinase-dead PKR controls removed from **Fig. 3G**. Bacteria were grown in M9 minimal medium supplemented with antibiotics and either induced or uninduced with IPTG with shaking for ≤30 hours at 37 °C. The *P* values are the likelihood of equivalent turbidity of *E. coli* transfected with the indicated constructs uninduced or induced with IPTG as calculated by unpaired student's t-test (n=7).

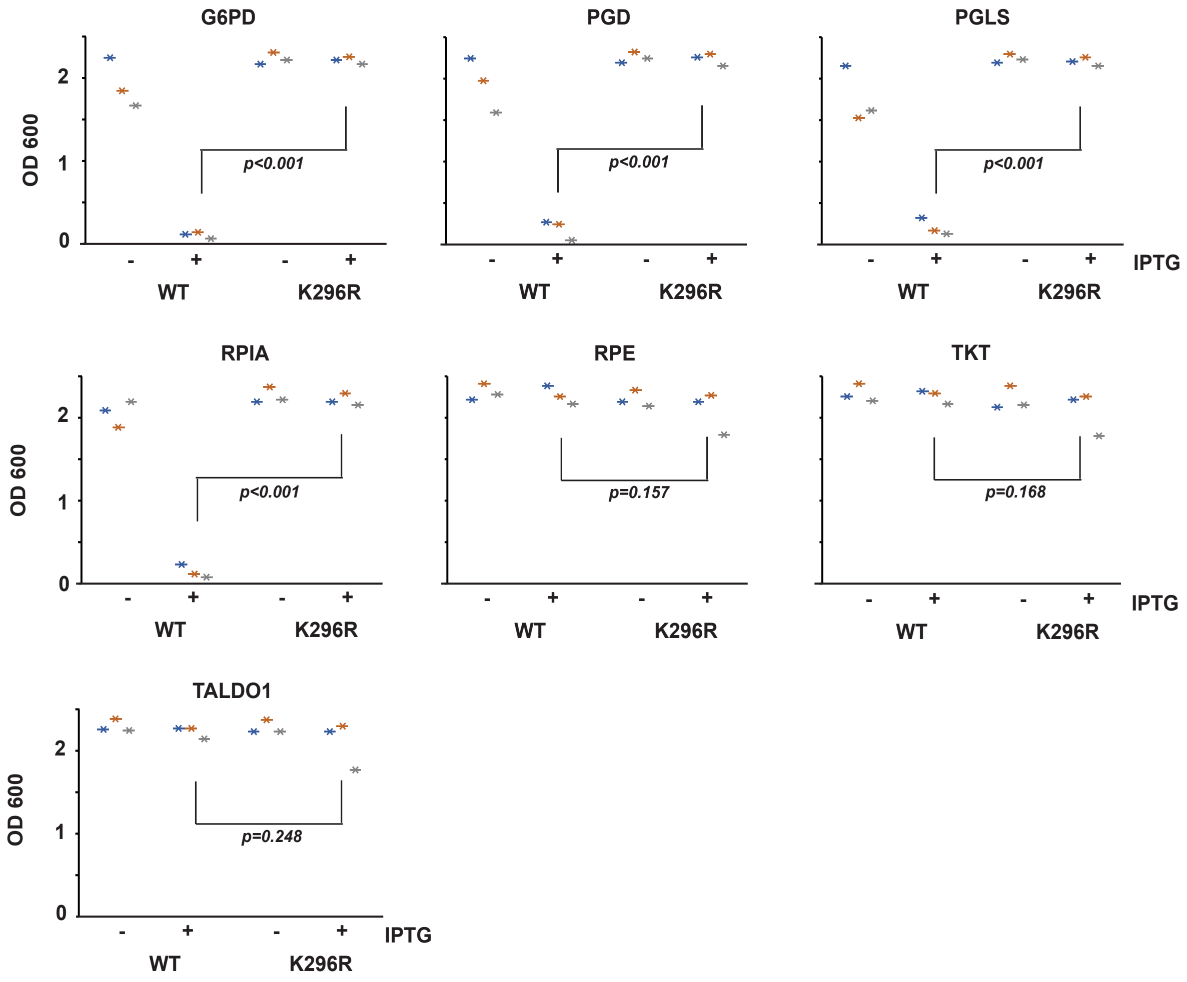

Appendix Figure S7

Supporting material for PKR's regulation of the human PPP proteins in *E. coli*. Plots of the growth of *E. coli* BL21(DE3)-pLysS-*pET15b-EIF2AK2* expressing WT or kinase-dead (K296R) PKR alone or co-transfected with *pWKS130* expressing the indicated PPP genes from humans by measures of the culture optical density (600 nm). The data shows the matched kinase-dead PKR controls removed from **Fig. 4A**. Bacteria were grown in an M9 minimal medium supplemented with antibiotics and either induced or uninduced with IPTG with shaking for ≤30 hours at 37 °C. The *P* values are the likelihood of equivalent turbidity of *E. coli* transfected with the indicated constructs uninduced or induced with IPTG as calculated by unpaired student's *t*-test of independent experiments (n=3).

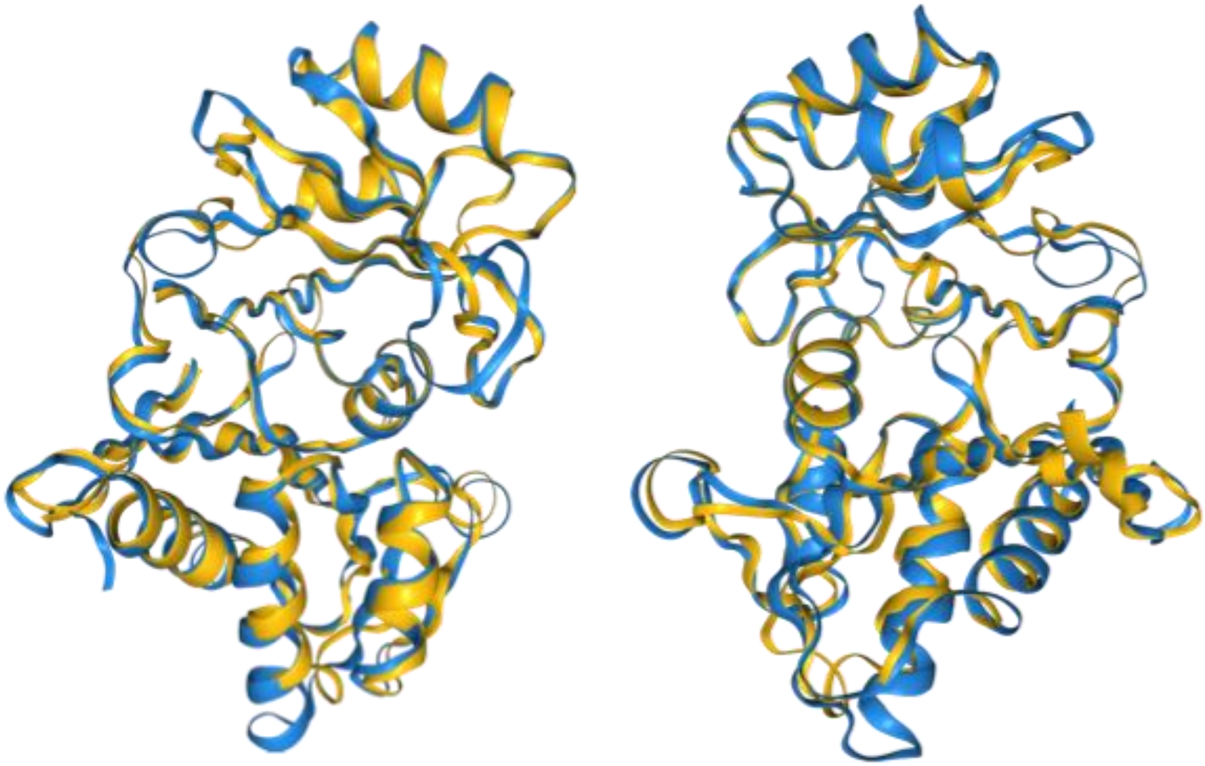

Appendix Figure S8

Comparison of the human and bacterial Ribose 5-phosphate isomerases. Superimposition of the ribbon diagrams of the ternary complex of Ribose 5-phosphate isomerases from *E. coli* (from the PDB file 1LKZ, in gold) and humans (from the Alpha fold model P49247-F1, in blue) with the 77 amino-terminal residues removed. Opposing faces of the proteins are shown on the left and right. Calculation of the Template Model (TM) as 0.94141 and the root-mean-square deviation of the average distance between atoms (RMSD) as 1.49 Å rank the protein folds as being highly homologous. This comparison was generated by Foldseek (https://foldseek.com). A PDB file of the superimposed proteins is supplied as Source Data 7. This analysis supports the use of the *E. coli* enzyme as a template for the human isomerase in the computational modelling that is shown in **Fig. 7A**. The conserved ternary structures with analysis of the protein's association in **Appendix Figure S11** also supports the proposition of the *E. coli* enzyme as a surrogate substrate in experiments shown in **Figs. 3D-G, 4A-D, 5A** and **6C-E**.

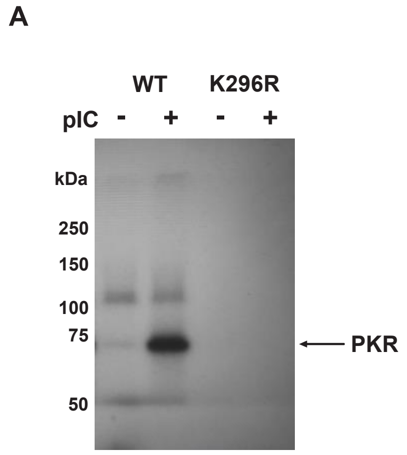

Appendix Figure S9

Supporting material for in vitro kinase assays. Autoradiographs detecting phosphoproteins as γP^{32}-labelled products that have been electrophoretically resolved by SDS-PAGE. (**A**) Radiolabeled products from in vitro kinase assays with the recombinant purified WT or kinase-dead (K296R) PKR proteins confirm the induction of catalytic activity by polyinosinic-polycytidylic acid (pIC) as autophosphorylated products. Monomeric PKR appears as a strongly radiolabeled signal at 68 kDa after activation. Minor signals are lower molecular weight cleaved products and a higher molecular weight active dimer that is unresponsive. (**B**) Radiolabeled products from in vitro kinase assays containing polyinosinic-polycytidylic acid and the WT PKR with EIF2S1/EIF2α, as a positive control, and two separate preparations of FLAG-tagged RPIA or RPIA without PKR to confirm the PKR-specific phosphorylation of RPIA. Notably, the signal is muted in '**B**' as the higher concentration of polyacrylamide prevents gel drying and necessitates exposure of the hydrated gel wrapped in plastic, as opposed to the direct exposure of the dried gel to film in '**A**'. Additionally, the FLAG-tagged proteins were immunoprecipitated from the kinase assays with an anti-FLAG antibody in '**B**' to minimise interference from autophosphorylated PKR peptides. These data support the direct phosphorylation of RPIA by PKR which is shown in **Fig. 5E**.

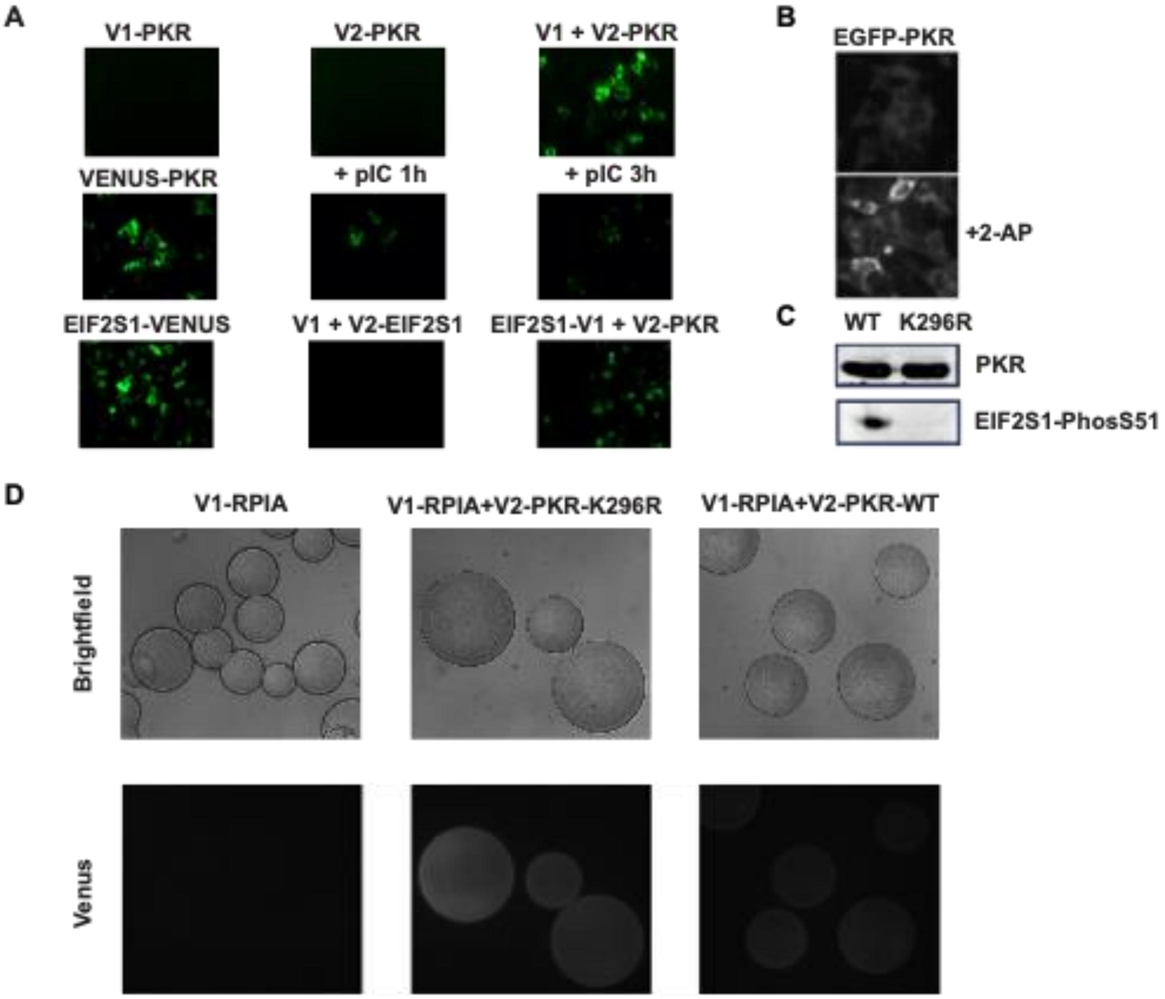

E

V1-RPIA
GGRTMVSKGEELFTGVVPILVELDGDVNGHKFSVSGEGEGDATYGKLTLKLICTTGKLPVPWPTLVTTL
GYGLQCFARYPDHMKQHDFFKSAMPEGYVQERTIFFKDDGNYKTRAEVKFEGDTLVNRIELKGIDFKE
DGNILGHKLEYNYNSHNVYITADKQIDGGGGSGGGGSSGMQRPGPFSTLYGRVLAPLPGRAGGAASG
GGGNSWDLPGSHVRLPGRAQSGTRGGAGNTSTSCGDSNSICPAPSTMSKAEEAKKLAGRAAVENHV
RNNQVLGIGSGSTIVHAVQRIAERVKQENLNLVCIPTSFQARQLILQYGLTLSDLDRHPEIDLAIDGADEV
DADLNLIKGGGGCLTQEKIVAGYASRFIVIADFRKDSKNLGDQWHKGIPIEVIPMAYVPVSRAVSQKFGG
VVELRMAVNKAGPVVTDNGNFILDWKFDRVHKWSEVNTAIKMIPGVVDTGLFINMAERVYFGMQDG
SVNMREKPFCDYKDDDDK*

V2-PKR
GGRTMKNGIKANFKIRHNIEDGGVQLADHYQQNTPIGDGPVLLHLSQSASPNEKRVLLIDLYKIDGGG
GSGGGGSSGMAGDLSAGFFMEELNTYRQKQGVVLKYQELPNSGPPHDRRFTFQVIIDGREFPEGEGR
SKKEAKNAAAKLAVEILNKEKKAVSPLLLTTTNSSEGLSMGNYIGLINRIAQKKRLTVNYEQCASGVHGP
EGFHYKCKMGQKEYSIGTGSTKQEAKQLAAKLAYLQILSEETSVKSDYLSSGSFATTCESQSNSLVTSTLA
SESSSEGDFSADTSEINSNSDSLNSSSLLMNGLRNNQRKAKRSLAPRFDLPDMKETKYTVDKRFGMDF
KEIELIGSGGFGQVFKAKHRIDG**K**TYVIKRVKYNNEKAEREVKALAKLDHVNIVHYNGCWDGFDYDPE
TSDDSLESSDYDPENSKNSSRSKTKCLFIQMEFCDKGTLEQWIEKRRGEKLDKVLALELFEQITKGVDYI
HSKKLIHRDLKPSNIFLVDTKQVKIGDFGLVTSLKNDGKRTRSKGTLRYMSPEQISSQDYGKEVDLYALG
LILAELLHVCDTAFETSKFFTDLRDGIISDIFDKKEKTLLQKLLSKKPEDRPNTSEILRTLTVWKKSPEKNER
HTC*

Appendix Figure S10

Supporting material for split Venus assays. (**A**) Micrographs of HEK293 cells expressing split Venus constructs. The Venus signals identify; (top) PKR dimerisation as fluorescence in cells expressing PKR monomers separately tagged with the different halves of Venus (V1 or V2) alone or together; (centre) PKR-dependent suppression of translation as reduced fluorescence from PKR tagged with the full-length fluorophore after treatment with polyinosinic-polycytidylic acid (pIC) and; (bottom) the association between PKR and EIF2S1/EIF2α as the relative fluorescent signal in cells expressing EIF2S1 tagged with the full-length fluorophore compared to molecules separately tagged with the split Venus as noninteracting EIF2S1 or EIF2S1 and PKR. (**B**) Micrographs of HEK293 transfected with EGFP-tagged PKR confirm the relief of translation control by PKR through increased fluorescence after the treatment with the kinase inhibitor 2AP (5 mM). (**C**) Detecting the expression and activity of WT and kinase-dead PKR in HEK293 cells by immunoblot with antibodies for PKR and the phosphorylated serine residue number 51 on EIF2S1/EIF2α. (**D**) Micrographs of GFP-Trap nanobody-coupled agarose beads (ChromoTek) incubated with lysates of HEK293 cells expressing the indicated split-Venus tagged constructs as brightfield (a top) and fluorescent images (below). (**E**) The amino acid sequence of the split-Venus tagged (coloured text) RPIA and PKR proteins (black text). The lysine number 296 that is mutated to produce the kinase-dead PKR is indicated in bold text. These data support the analysis of the association between PKR and RPIA and the detection of phosphoresidues on RPIA shown in **Fig. 6A-D** and **Table 1**.

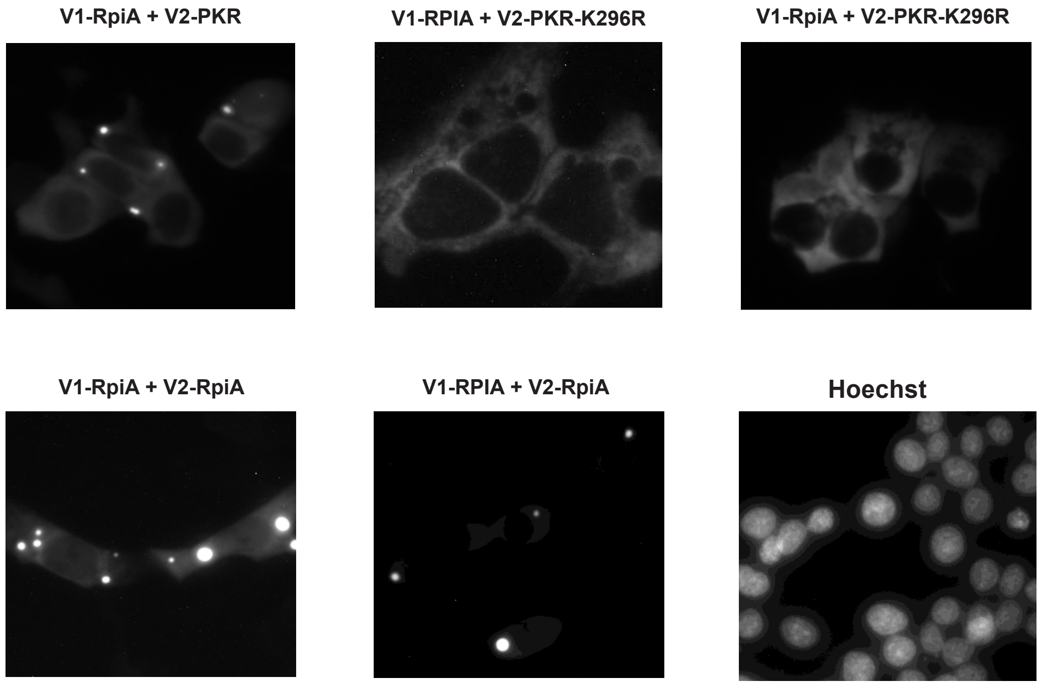

Appendix Figure S11

Material supporting PKR's control of the *E. coli* isomerase. Micrographs of HEK293 cells expressing split Venus constructs. The Venus signals identify; (top) the interaction between PKR and the human RPIA (centre and **Fig. 6A** and **B** and modelled in **Fig. 7A**) is retained with the *E. coli* isomerase (top left and right) and identifies that the propensity of the isomerase to oligomerise (bottom left and **Fig. 6C**) extends to hetero-oligomeric complexes (bottom centre). The nuclei of the cells co-expressing split Venus-tagged human and *E. coli* isomerases are visualised by Hoechst staining (bottom right). These data identify functional conservation of the isomerase that is predicted by the primary and ternary structures (**Fig. 6D** and **Appendix Figure S8**) that support the assertion that RpiA is a surrogate PKR substrate, as demonstrated in **Figs. 3D-G**, **4A-D** and **7C-E**. As well as evidence for the similarity of the *E. coli* and human isomerases, the association between RPIA and RpiA may also explain a faint 27 kDa phosphoprotein that was immune-enriched from the lysates of transformed *E. coli* with the FLAG-tagged RPIA by an anti-FLAG antibody (**Fig. 5A** and **Appendix Figure S3**).

Appendix Table S1

The metabolic enzymes cloned as mobile plasmids (*pNTR-SD* or *pCA24N*) in *E. coli* F+ JA200 donor strains that were matted with the recipient BL21(DE3) pLysS/*pET15b-EIF2AK2* to identify the metabolic defect induced by PKR's kinase activity (**Fig. 3F** and **Appendix Figure S5 & S6**).

**Enzyme**	**Gene**	**Activity**
Adenylate kinase	*Adk*	Ribonucleotide biosynthesis
Glutamate/aspartate ABC transporter	*GltL*	Transport of L-aspartate/glutamate
Cysteine synthase A	*CysK*	L-cysteine metabolism
Translation elongation factor Tu 1	*TufA*	Protein translation
Maltose ABC transporter periplasmic binding protein	*MalE*	Transport of malt sugar
Acetyl CoA synthetase (AMP-forming)	*Acs*	Activates acetate
**β-hydroxyacyl-[acyl] dehydratase/isomerase**	***FabA***	**Fatty acid biosynthesis**
**3-oxoacyl-[acyl carrier protein] synthase 3**	***FabH***	**Fatty acid biosynthesis**
**[acyl-carrier-protein] *S*-malonyltransferase**	***FabD***	**Fatty acid biosynthesis**
DNA-binding transcriptional dual regulator	*FadR*	Fatty acid metabolism
**Enoyl-[acyl-carrier-protein] reductase**	***Fabl***	**Fatty acid biosynthesis**
**3-Oxoacyl-[acyl carrier protein] synthase 1**	***FabB***	**Fatty acid biosynthesis**
**Acetyl-CoA carboxyltransferase subunit α**	***AccA***	**Fatty acid biosynthesis**
**Acyl carrier protein**	***AcpP***	**Fatty acid biosynthesis**
**3-Hydroxy-[acyl] dehydratase**	***FabZ***	**Biotin and fatty acid biosynthesis**
3-Oxoacyl-[acyl] synthase 2	*FabF*	Biotin and fatty acid biosynthesis
**Acetyl-CoA carboxyltransferase subunit β**	***AccD***	**Biotin and fatty acid biosynthesis**
**Biotin carboxyl carrier protein**	***AccB***	**Biotinylation**
**Biotin carboxylase**	***AccC***	**Biotinylation**
Citrate synthase	*GltA*	Citric acid cycle
Succinate: quinone oxidoreductase C	*SdhC*	Citric acid cycle
Succinate: quinone oxidoreductase D	*SdhD*	Citric acid cycle
Succinate: quinone oxidoreductase B	*SdhB*	Citric acid cycle
2-Oxoglutarate dehydrogenase, decarboxylase	*SucA*	Citric acid cycle
2-Oxoglutarate dehydrogenase, succinyltransferase	*SucB*	Citric acid cycle
Succinyl CoA synthetase subunit α	*SucD*	Citric acid cycle
Aconitate hydratase A	*AcnA*	Citric acid cycle
Fumarase C	*FumC*	Citric acid cycle
Fumarase A	*FumA*	Citric acid cycle
Fumarase B	*FumB*	Citric acid cycle
Pyruvate dehydrogenase, E1 subunit	*AceE*	Glucose metabolism
Pyruvate dehydrogenase, E2 subunit	*AceF*	Glucose metabolism
Lipoamide dehydrogenase	*LpdA*	Glucose metabolism
Fructose-bisphosphate aldolase class I	*FbaB*	Glucose metabolism
Fructose-bisphosphate aldolase class II	*FbaA*	Glucose metabolism
**Fructose-1,6-bisphosphatase 1**	***Fbp***	**Glucose metabolism**
Enolase	*Eno*	Glucose metabolism
Phosphoglycerate kinase	*Pgk*	Glucose metabolism
Fructose 1,6-bisphosphatase	*YggF*	Glucose metabolism
Triose-phosphate isomerase	*TpiA*	Glucose metabolism
Fructose-1,6-bisphosphatase 2	*GlpX*	Glucose metabolism
6-Phosphofructokinase 1	*PfkA*	Glucose metabolism
2,3-Bisphosphoglycerate-dependent phosphoglycerate mutase	*GpmA*	Glucose metabolism
**Pyridoxal phosphate phosphatase**	***YbhA***	**Glucose metabolism**
Pyruvate kinase 1	*PykF*	Glucose metabolism
Pyruvate kinase 2	*PykA*	Glucose metabolism

**6-Phosphofructokinase 2**	***PfkB***	**Glucose metabolism**
Glyceraldehyde-3-phosphate dehydrogenase	*GapA*	Glucose metabolism
**Glucose-6-phosphate isomerase**	***Pgi***	**Glucose metabolism**
Zwischenferment	*Zwf*	Glucose metabolism
6-Phosphogluconate dehydrogenase	*Gnd*	Glucose metabolism
Phosphoenolpyruvate synthetase	*PpsA*	Glucose metabolism
**Malate dehydrogenase**	***Mdh***	**Glucose metabolism**
Fructose 1,6-bisphosphatase 2	*Glpx*	Glucose metabolism
Putative phosphatase	*GpmB*	Carbohydrate metabolism
**Ribose isomerase B**	***RpiB***	**Carbohydrate metabolism**
**Transketolase**	***TktA***	**Carbohydrate metabolism**
Transketolase	*TktB*	Carbohydrate metabolism
*Transaldolase	*TalB*	Carbohydrate metabolism
Ribose 5-phoshate isomerase	*RpiA*	Carbohydrate metabolism

*After purification the open reading frame of the *TalB* gene was found to contain a deletion.

Clones that reduced the auxotrophic effects of PKR are bolded.

These clones were sourced from the National BioResource Project: *E. coli* Microbial Genetics Laboratory, Genetic Strains Research Center, Research Organization of Information and Systems, National Institute of Genetics, 1111Yata, Mishima, Shizuoka, 411-8540 Japan.
