# Supplementary figures and images for: The immune response to RNA suppresses nucleic acid synthesis by limiting ribose 5-phosphate
Source: EMBO J. 2024 May 22;43(13):2636–60. doi: 10.1038/s44318-024-00100-w (PMC11217295; doi:10.1038/s44318-024-00100-w)

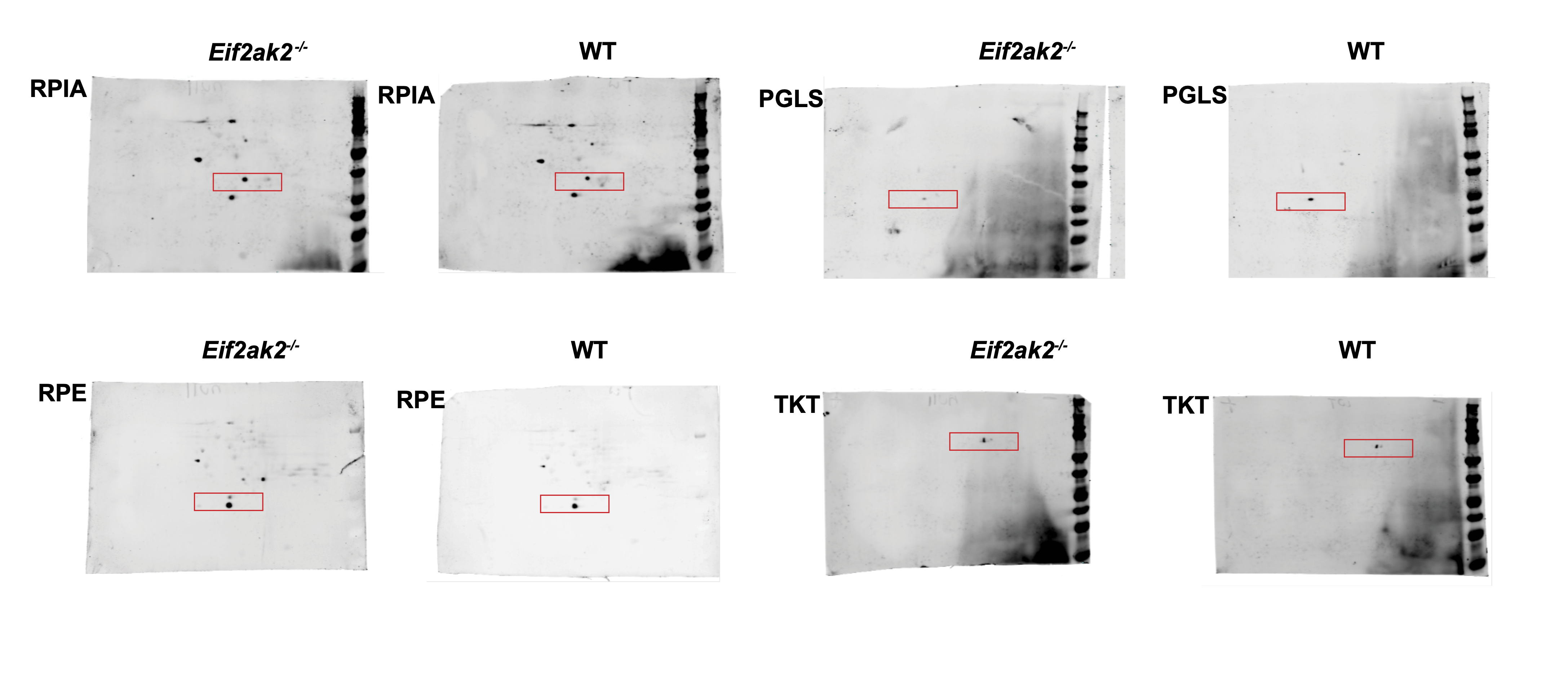

Supplement: Supplementary file 6 — Source data Fig. 5 [file 44318_2024_100_MOESM6_ESM.zip › SD5/Image Fig 5B.tif]

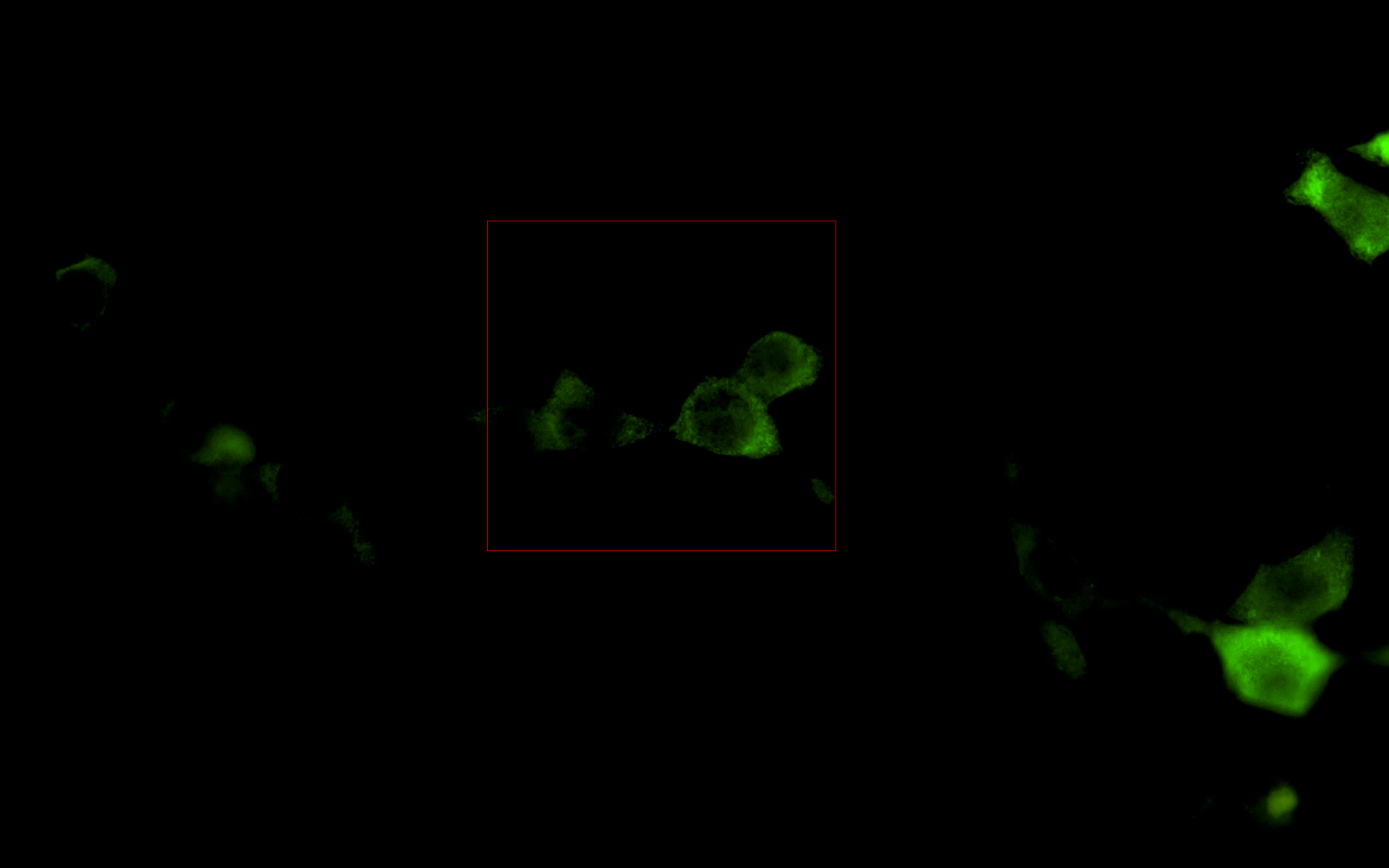

Supplement: Supplementary file 7 — Source data Fig. 6 [file 44318_2024_100_MOESM7_ESM.zip › SD6/Image Fig 6A.tiff]

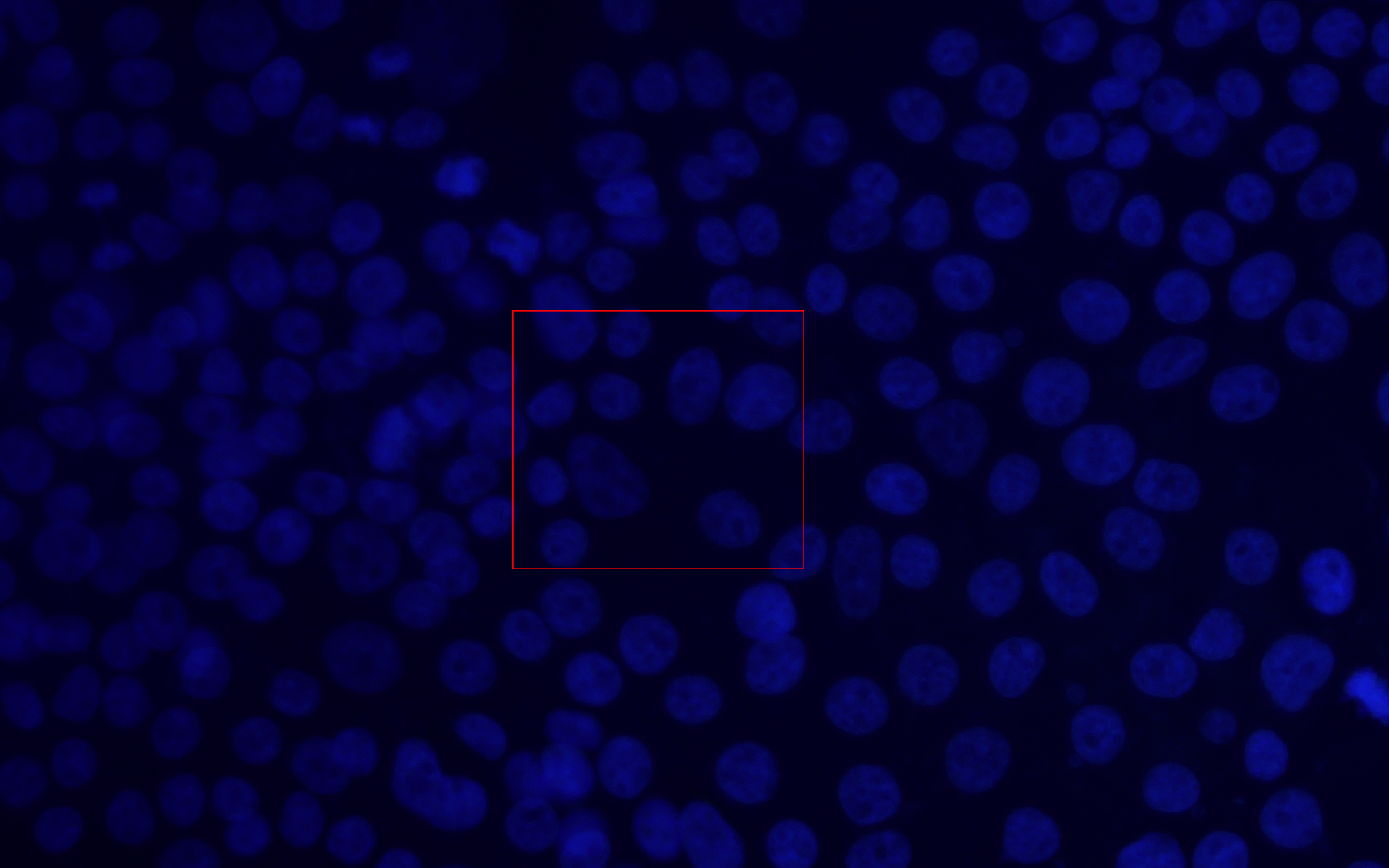

Supplement: Supplementary file 7 — Source data Fig. 6 [file 44318_2024_100_MOESM7_ESM.zip › SD6/Image Fig 6Cii.tif]

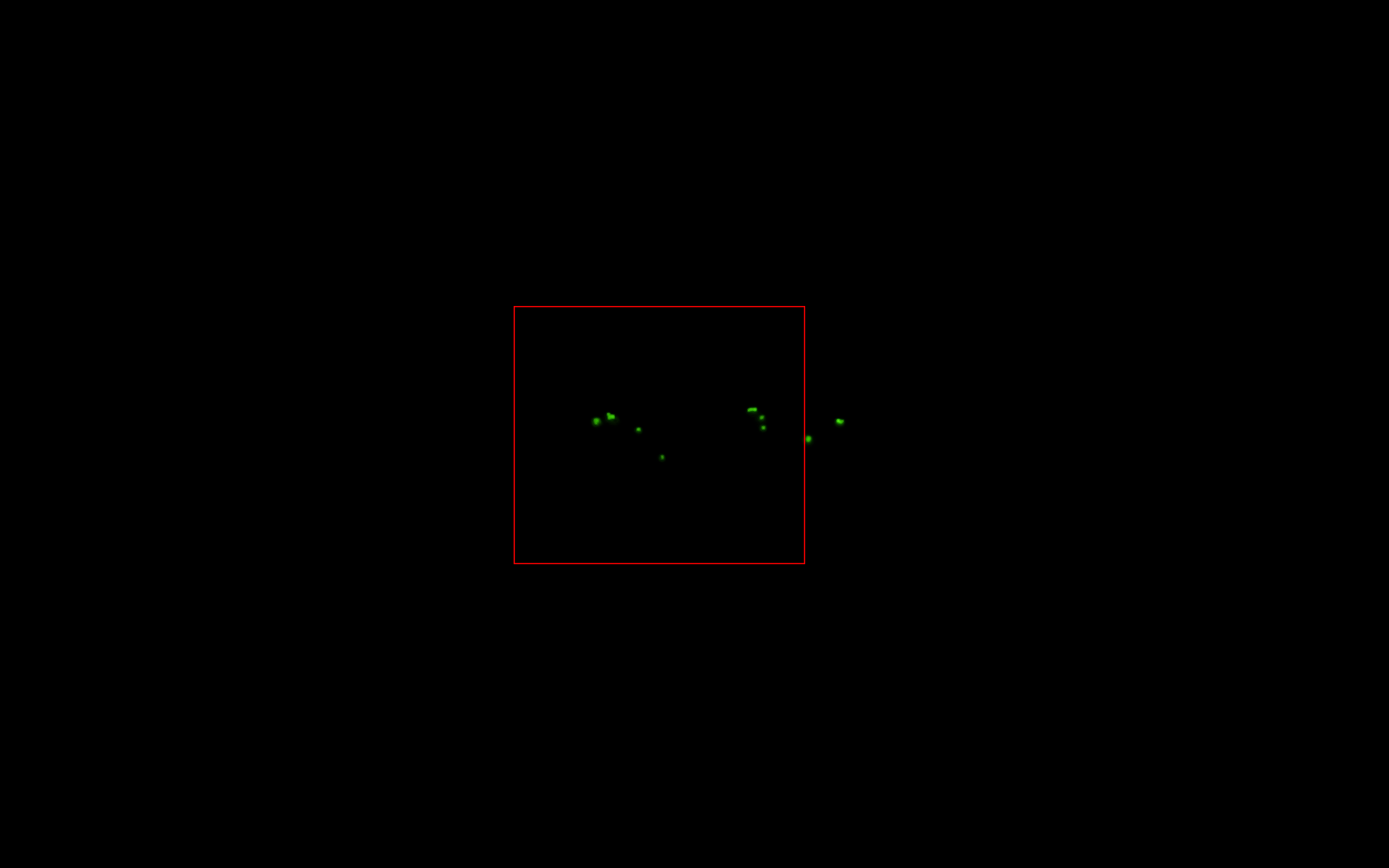

Supplement: Supplementary file 7 — Source data Fig. 6 [file 44318_2024_100_MOESM7_ESM.zip › SD6/Image Fig 6Ci.tif]

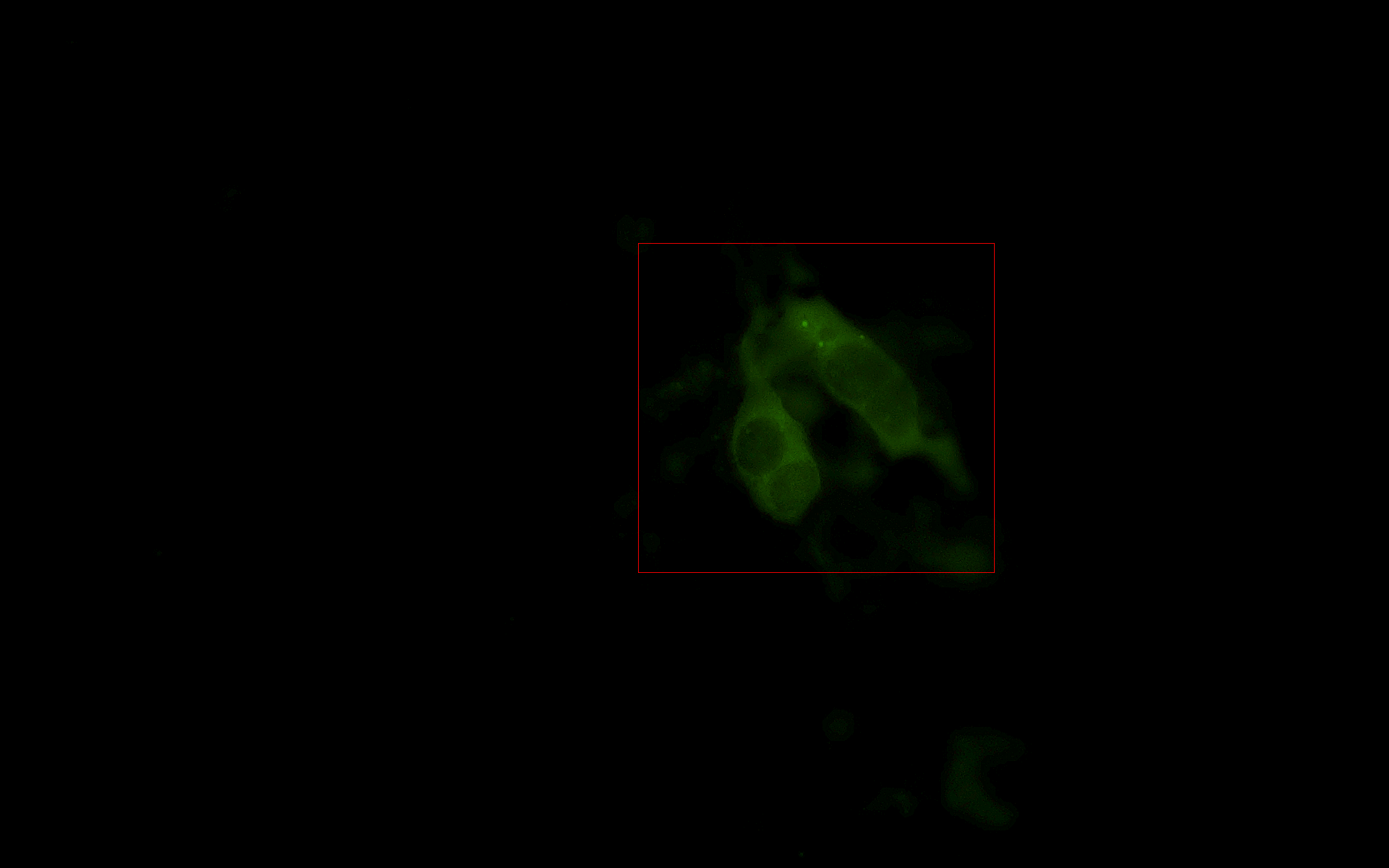

Supplement: Supplementary file 7 — Source data Fig. 6 [file 44318_2024_100_MOESM7_ESM.zip › SD6/Image Fig 6B.tiff]

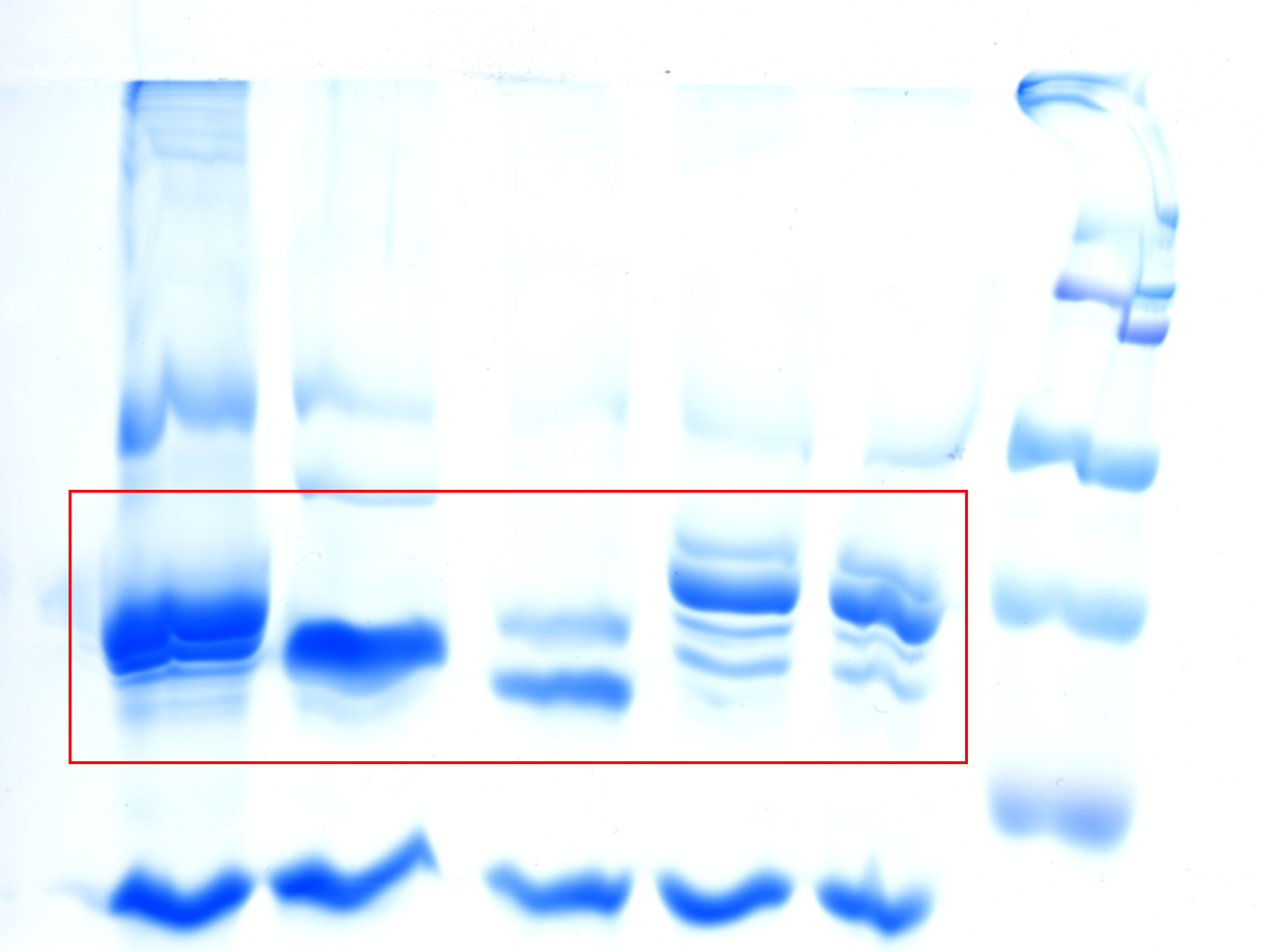

Supplement: Supplementary file 8 — Source data Fig. 7 [file 44318_2024_100_MOESM8_ESM.zip › SD7/Image Fig 7B.tif]

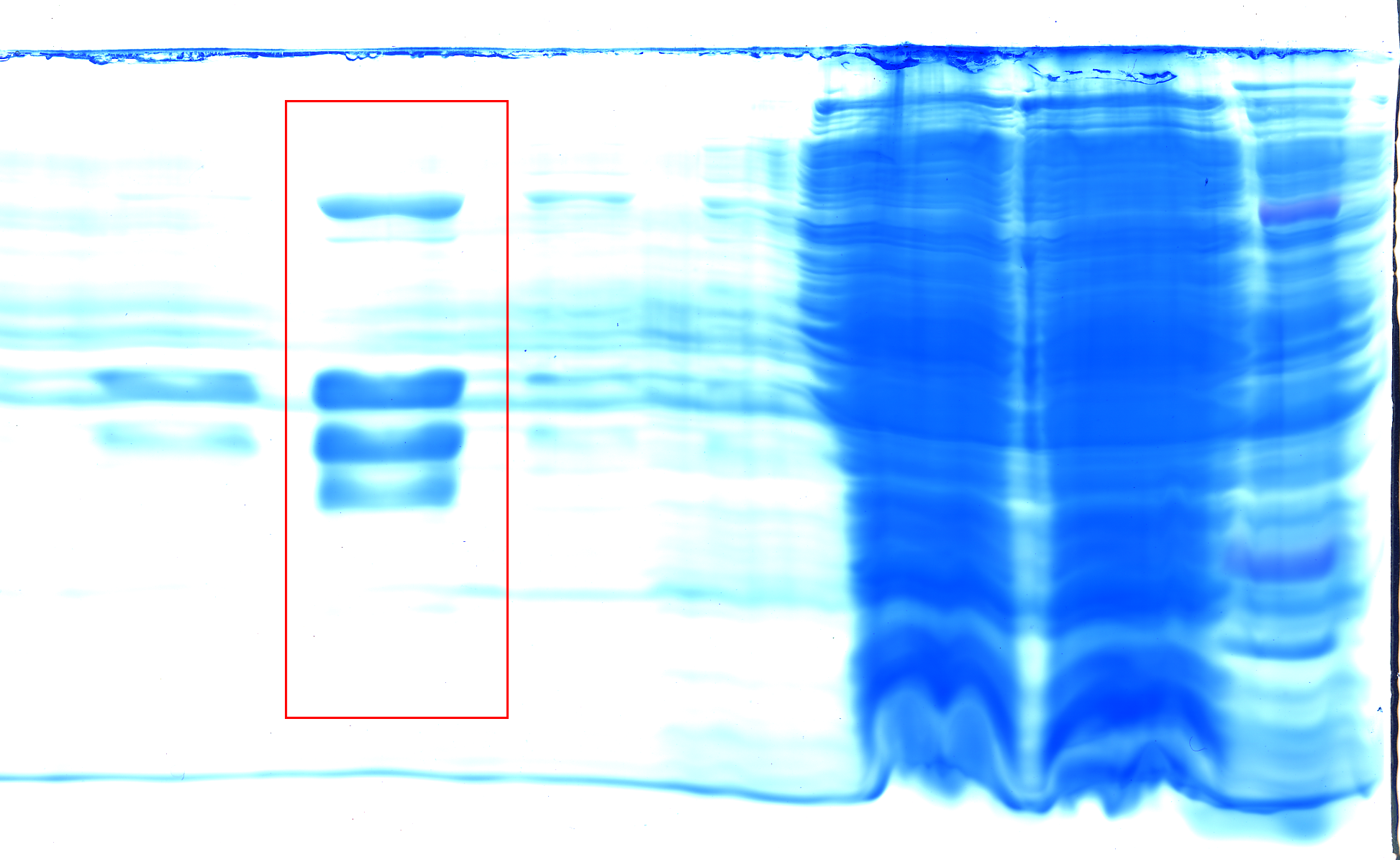

Supplement: Supplementary file 9 — Source data Fig. 8 [file 44318_2024_100_MOESM9_ESM.zip › SD8/Image Fig 8B.tif]
